# Synthesis and characterization of cobalt SCS pincer complexes

**DOI:** 10.1007/s00706-022-02949-1

**Published:** 2022-07-16

**Authors:** Jan Pecak, Matthias Käfer, Sarah Fleissner, Werner Artner, Karl Kirchner

**Affiliations:** 1 Institute of Applied Synthetic Chemistry, Vienna University of Technology, Getreidemarkt 9/163-AC, 1060 Vienna, Austria; 2 X-Ray Center, Vienna University of Technology, Getreidemarkt 9/163-AC, 1060 Vienna, Austria

**Keywords:** Pincer complex, Cobalt, DFT, Transmetalation

## Abstract

The synthesis and characterization of two Co(II) complexes stabilized by a tridentate SCS pincer ligand are described. Paramagnetic [Co(κ^3^-SCS^CH2^-Et)_2_] and [Co(κ^3^-SCS^CH2^-*t*Bu)(κ^2^-SCS^CH2^-*t*Bu)] were obtained via transmetalation protocol from CoBr_2_ and S(C-Br)S^CH2^-R (R = Et, *t*Bu). Oxidation of the latter with [Cp_2_Fe]PF_6_ affords the diamagnetic 18 VE complex [Co(κ^3^-SCS^CH2^-*t*Bu)_2_]PF_6_. X-ray structures and DFT calculations are presented.

**Graphical abstract:**

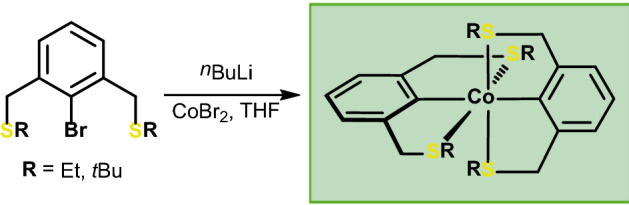

**Supplementary Information:**

The online version contains supplementary material available at 10.1007/s00706-022-02949-1.

## Introduction

In the last decades, pincer ligands evolved to be extremely versatile scaffolds for the stabilization of transition metal fragments. Whereas the chemistry of PCP pincer complexes with first row transition metals is well established, reports on corresponding SCS systems are scarce [1–4]. To the present date, aryl-based SCS pincer complexes with thioether donor groups are only known for Ni, Pt, and Pd. The first κ^3^-SCS Pd system was prepared by Dupont et al. via transcylometalation of S(C-H)S^CH2^-Me with [{Pd(C_6_H_4_CH_2_NMe_2_)(μ-Cl)}_2_] in boiling benzene [5]. Later, van Koten and co-workers synthesized numerous corresponding compounds by reacting the ligand S(C-Br)S^CH2^-R (R = Me, Ph) with appropriate precursors, such as [Ni(COD)_2_] or [Pd_2_dba_3_] at ambient temperature [1, 6]. Regarding κ^3^-SCS complexes with thioamide moieties, numerous complexes are known for Pt, Pd, and Ni [7, 8]. Following the HSAB concept, both phosphine and thioether donors can be described as rather soft ligands. Accordingly, the calculated chemical hardness η for PH_3_ is comparable to H_2_S (5 eV), whereas the value for NH_3_ (6.9 eV) is in line with a description as hard ligand [9]. Moreover, thiolates tend to act as bridging ligands which can result in the formation of oligomeric compounds or clusters. As far as cobalt is concerned, stable κ^3^-type PCP, NCN, PNP, and SNS pincer complexes are well known to literature but no SCS systems [10–14]. In this contribution, we report on the synthesis and characterization of three new cobalt SCS pincer complexes. X-ray structures and DFT calculation are presented.

## Results and discussion

**Fig. 1:**
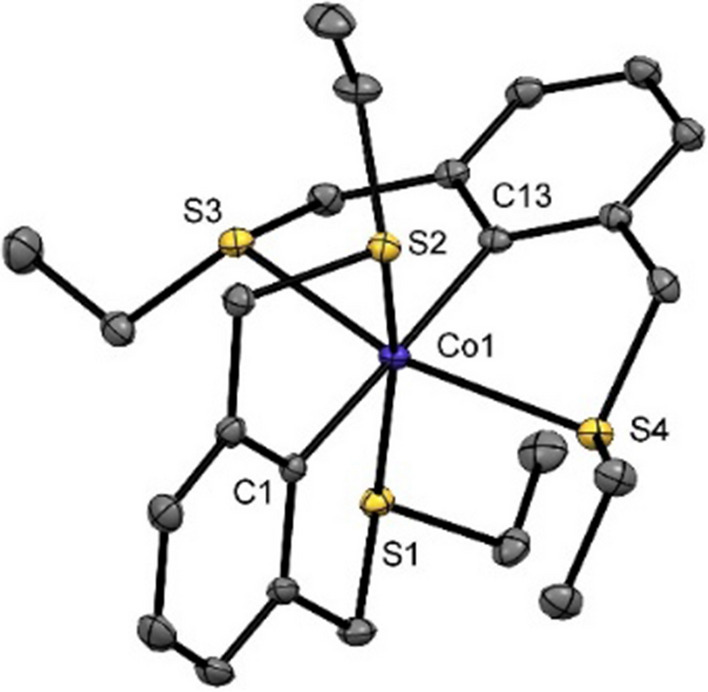
Structural view of **1** showing 50% displacement ellipsoids (H atoms omitted for clarity). Selected bond lengths [Å] and angles [°]: Co1-C1 1.9570(16), Co1-C13 1.9575(16), Co1-S1 2.3021(5), Co1-S2 2.2855(5), Co1-S3 2.4253(5), Co1-S4 2.4771(5), C1-Co1-C13 177.56(6), C1-Co1-S1 82.38(5)

Based on our recent studies on cobalt NCN and PCP pincer complexes, we envisioned to prepare analogous SCS complexes via similar protocols. Lithiation of 2-bromo-1,3-bis[(ethylthio)methyl]benzene with *n*BuLi at – 90 ℃ and subsequent treatment of this solution with CoBr_2_ (or CoCl_2_) suspended in THF at 0 ℃ led to a color change from initially light yellow to dark red. After extraction of the solid residue with pentane, compound **1** could be isolated in 24% yield (Scheme 1). To establish the solid-state structure, single crystals were grown from a saturated solution in pentane kept in a freezer. An ORTEP representation of the molecular structure is depicted in Fig. 1 with selected metrical parameters reported in captions. The effective magnetic moment of **1** was consecutively measured using the Evans method (*μ*_eff_ = 1.9 *μ*_B_, C_6_H_6_) in solution and is consistent with a paramagnetic Co(II) complex that contains one unpaired electron.

**Figure:**
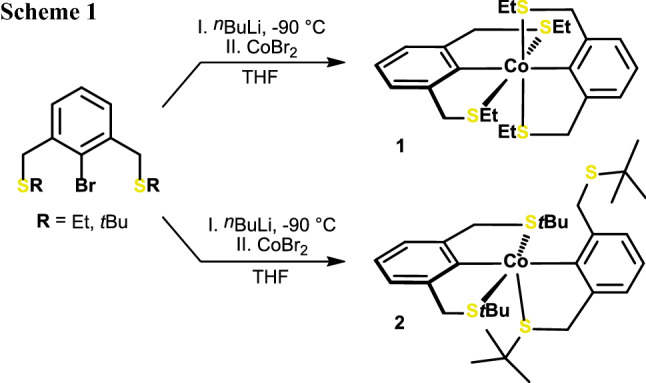

Contrary to our expectations, no square-planar pincer complex of the form [Co(SCS^CH2^-Et)Br] was obtained in analogy to recently reported [Co(NCN^CH2^-Et)Br], but a distorted octahedral 19 VE κ^3^κ^3^-type complex together with significant amounts of intractable material (vide infra). The mean Co-C bond length is 1.961(2) Å and therefore comparable to [Co(κ^3^-PCP^NMe^-*i*Pr)Cl] (cf. 1.92 Å) but significantly longer than in [Co(κ^3^-NCN^CH2^-Et)Br] (cf. 1.85 Å) [10, 12]. The Co-S bond lengths are surprisingly unequal in both κ^3^-units with mean values of 2.3040(8) Å and 2.4323(5) Å, respectively. The C1-Co-C13 bond angle is 177.56(6)°, and the molecule is almost S_4_ symmetric.

**Fig. 2:**
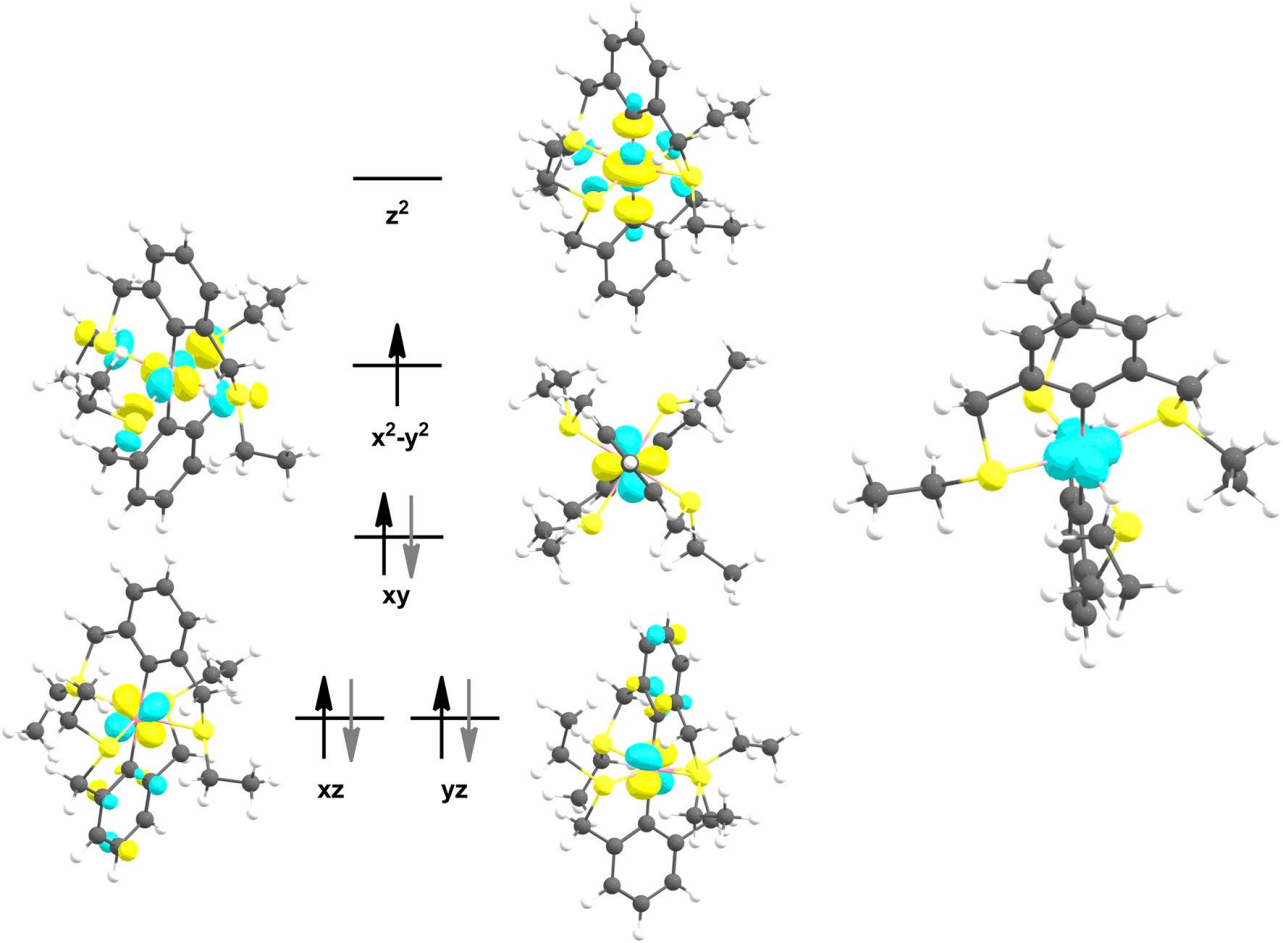
DFT-calculated frontier orbitals of [Co(κ^3^-SCS^CH2^-Et)_2_] (BP86/def2-SVP) and spin density plot

To investigate the electronic structure of compound **1**, DFT calculations were performed. [Co(SCS^CH2^-Et)_2_] was optimized on a full model for its doublet and quartet spin state at the BP86-D3/def2-SVP level of theory. The calculations revealed that the low-spin state is 138 kJ/mol (and 49.4 kJ/mol with PBE0) more stable than the high-spin case (*S* = 3/2), in line with experiments. The low-spin structure agrees favorably with experimental values, whereas the high-spin structure deviates strongly with a C–Co-C angle of 159.6° and mean Co-S distances of 2.52(8) Å. A molecular structure specified with C–Co-C = 180.0° is only 0.67 kJ/mol less stable than the fully relaxed one. Figure 2 displays the qualitative d-splitting for complex **1** with the d_x2-y2_ orbital being the SOMO and the d_z2_ being the LUMO in accordance with an octahedral d^7^ system. Due to the high molecular symmetry, the d_xz_ and d_yz_ orbitals are energetically degenerate.

**Fig. 3:**
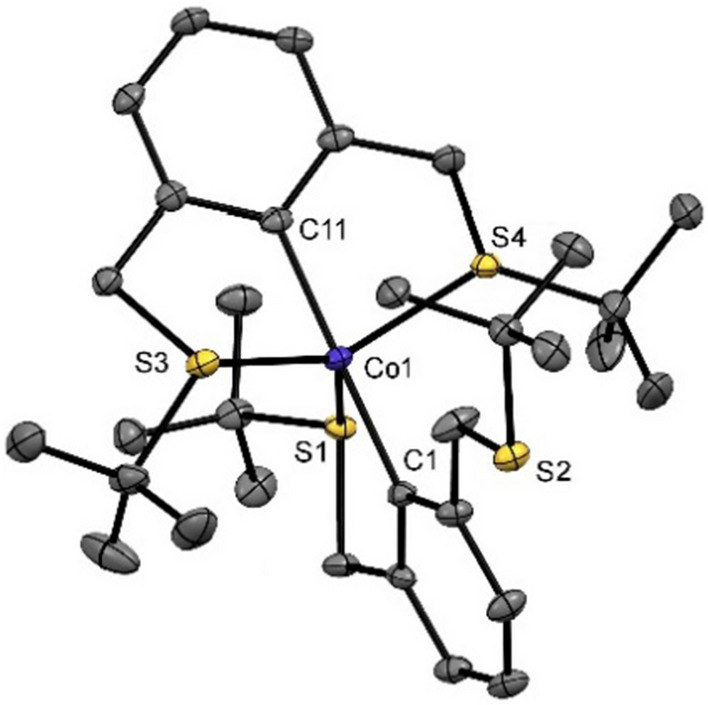
Structural view of **2** showing 50% displacement ellipsoids (H atoms omitted for clarity). Selected bond lengths [Å] and angles [°]: Co1-C1 1.992(3), Co1-C11 1.985(3), Co1-S1 2.3185(8), Co1-S3 2.2257(8), Co1-S4 2.2553(8), C1-Co1-C11 177.67(11), S3-Co1-S4 135.11(3), S3-Co1-S1 124.72(3)

In order to impose κ^3^-type coordination and to avoid twofold transmetalation, the more sterically demanding 2-bromo-1,3-bis[(*tert*-butylthio)methyl]benzene was prepared as a ligand. Applying an analogous synthetic protocol and extraction of the solid residues with pentane afforded a dark yellow to red compound in low yield. Single crystals of **2** suitable for X-ray diffraction were obtained from a saturated pentane solution kept at – 30 ℃. A view of the molecular structure is depicted in Fig. 3 with selected bond distances and angles reported in captions. The 17 VE complex adopts a distorted trigonal bipyramidal (*τ*_5_ = 0.71) [15] structure with the metal center coordinated in a κ^2^κ^3^ fashion and one pending CH_2_S*t*Bu arm. The lower coordination number in complex **2** is accompanied with a shortening of the Co-S distances as compared to compound **1**.

As described (vide supra), pentane was used to extract **2** from the solid residue afforded by the transmetalation protocol. Interestingly, the remaining residue can be further extracted into toluene to afford a dark green solid. This seemingly stable compound is soluble in polar solvents including MeOH and can be precipitated by addition of pentane. HRMS analysis in the positive ion mode gave evidence for the prevalence of a formally cationic** 2**^+^ with an intact molecular ion peak at *m/z* = 621.2138 (calc. *m/z* 621.2122). In the negative ion mode, various inconclusive cobalt bromo patterns were found that support the presence of a complex counterion and the assumption of an overall disproportionation process. To support this thesis, complex **2** was treated with [Cp_2_Fe]PF_6_ in solution to afford a dark green product (Scheme 2) [16]. This compound was identified as the symmetric Co(III) κ^3^κ^3^-type complex 3. Due to its diamagnetism, **3** could be characterized by ^1^H NMR spectroscopy as well as HRMS analysis. In the ^1^H NMR spectrum the C*H*_2_S*t*Bu (linker) protons give rise to a broad singlet at 4.23 ppm (cf 3.95 ppm in the free ligand). The broadness of the proton NMR signal likely originates from fluxional coordination behavior of the alkylthio donors in solution. The oxidation process clearly leads to the formation of a coordinatively saturated and most stable 18 VE complex.

**Figure:**
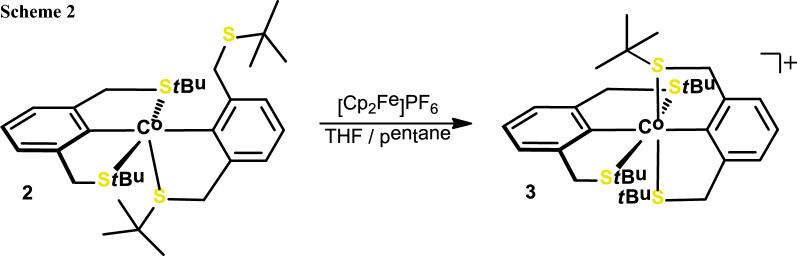

## Conclusion

The chemistry of cobalt pincer complexes has been dominated by strong-field ligands based on the PCP scaffold. Using SCS designs with SCS^CH2^-Et and SCS^CH2^-*t*Bu as ligands allowed for the synthesis of octahedral low-spin Co(II) complexes with κ^3^κ^3^ and κ^2^κ^3^ coordination modes. Upon oxidation with mild oxidants, a very stable 18 VE Co(III) complex is accessible.

## Experimental

All manipulations were performed under an inert atmosphere of argon by using Schlenk techniques or in an MBraun inert-gas glovebox. The solvents were purified according to standard procedures. The deuterated solvents were purchased from Aldrich and dried over 4 Å molecular sieves. ^1^H, ^13^C{^1^H}, and ^31^P{^1^H} NMR spectra were recorded on a Bruker AVANCE-400 spectrometer. ^1^H and ^13^C{^1^H} NMR spectra were referenced internally to residual protio-solvent and solvent resonances, respectively, and are reported relative to tetramethylsilane (*δ* = 0 ppm). The ligand S(C-Br)S^CH2^-Et was prepared according to the literature [17]. Infrared spectra were recorded in attenuated total reflection (ATR) mode on a PerkinElmer Spectrum Two FT-IR spectrometer. High resolution accurate mass spectra were recorded on an Agilent 6545 QTOF equipped with a dual electrospray ionization source (Agilent Technologies, Santa Clara, CA, USA). The mass calibration was performed with a commercial mixture of perfluorinated trialkyl-triazines. Elemental analysis was performed on an elementar vario MACRO (Elementar Analysensysteme GmbH, Germany) CHNS analyzer.

### 2-Bromo-1,3-bis[(*tert*-butylthio)methyl]benzene (SC(-Br)S^CH2^-tBu, C_16_H_25_BrS_2_)

2-Bromo-1,3-bis(bromomethyl)benzene (1.00 g, 2.91 mmol) was added to a solution of 0.63 g *tert*-butylmercaptane (7.00 mmol) and 0.350 g NaOH (8.73 mmol) in 10 cm^3^ dry methanol. The reaction mixture was stirred for 2 h at room temperature whereupon partial precipitation of sodium bromide was observed. Water (5 cm^3^) and 15 cm^3^ diethylether were added, and phases separated. The organic phase was washed with a 10% NaOH solution, dried over magnesium sulfate, and the solvent removed. Yield: 1.01 g (95%); ^1^H NMR (400 MHz, CD_2_Cl_2_): *δ* = 7.34 (*d*, *J* = 7.5 Hz, 2H, ph), 7.21 (*t*, *J* = 7.0 Hz, 1H, ph), 3.95 (s, 4H, CH_2_), 1.41 (s, 18H) ppm; ^13^C{^1^H} NMR (101 MHz, CD_2_Cl_2_): *δ* = 139.0, 129.6, 127.1, 126.4, 43.0, 34.4, 30.6 ppm; HRMS (ESI^+^, MeOH): *m/z* calc. for C_16_H_25_BrNaS_2_ ([M + Na]^+^) 383.0473, found 383.0475.

### Bis[κ^3^κ^3^-(2,6-bis(ethylthio-*κS*)methyl)phenyl-κC]cobalt(II) (1, C_20_H_26_CoS_4_)

To a solution of 0.135 g SC(-Br)S^CH2^-Et (0.44 mmol) in 8 cm^3^ THF/Et_2_O (1:1) was slowly added 0.30 cm^3^ *n*BuLi (1.6 M, 0.48 mmol) at – 90 ℃ and then stirred for 1 h. After allowing to warm to 0 ℃, a suspension of 0.100 g CoBr_2_ (0.44 mmol) in THF was added dropwise and very slowly. The reaction mixture was stirred for 20 min, and all volatiles were removed in vacuo. The remaining dark solid was extracted into pentane and the extract filtered through a syringe filter. The solvent was removed under reduced pressure to give a dark orange to red solid. Yield: 0.055 g (24%); *μ*_eff_ = 1.9 *μ*_B_ (benzene, Evans method).

### Bis[κ^3^κ^2^-(2,6-bis(*tert*-butylthio-*κS*)methyl)phenyl-κC]cobalt(II) (2, C_32_H_**50**_CoS_4_)

The synthesis was performed analogous to 1 with 0.150 g SC(-Br)S^CH2^-*t*Bu (0.41 mmol), 0.28 cm^3^ *n*BuLi (1.6 M, 0.45 mmol), and 0.090 g CoBr_2_ (0.41 mmol). Yield: 0.051 g (20%); *μ*_eff_ = 1.8 *μ*_B_ (CH_2_Cl_2_, Evans method).

## Reaction of 2 with [Cp_2_Fe]PF_6_—formation of 3 (C_32_H_50_CoS_4_PF_6_)

To a solution of 0.030 g complex 2 (0.04 mmol) in 5 cm^3^ pentane was dropwise added a solution of 0.014 g ferrocenium hexafluorophosphate (0.04 mmol) in 2 cm^3^ THF/CH_3_CN (5:1) until the initial color disappeared. During addition, a green substance precipitated that was eventually washed twice with pentane and dried in vacuum. ^1^H NMR (400 MHz, CD_2_Cl_2_): *δ* = 7.47 (*d*, *J* = 7.2 Hz, 4H, ph), 7.32 (*t*, *J* = 6.5 Hz, 2H, ph), 4.23 (bs, 8H, C*H*_2_StBu), 1.01 (s, 36H, CH_3_) ppm; ^13^C{^1^H} NMR (101 MHz, CD_2_Cl_2_): *δ* = 148.3, 126.7, 123.2, 52.2, 46.4, 29.8 ppm; HRMS (ESI^+^, MeOH): *m/z* calc. for C_32_H_50_CoS_4_ (M^+^) 621.2122, found 621.2138.

## Computational details

All calculations were performed using the ORCA 4.2.1 software package utilizing the Vienna Scientific Cluster (VSC3) in part [18]. Electronic ground state calculations, including geometry optimizations and frequencies, were carried out with density functional theory (DFT) using the functionals BP86 and PBE0, the def2-SVP basis set for the C, H, S atoms, and the def2-TZVP for cobalt [19, 20]. The D3 correction by Grimme together with BJ damping was used to account for dispersion interactions [21]. Orbital plots and graphics were generated with Chemcraft [22].

## X-Ray structure determination

X-ray diffraction data for** 1** and **2** (CCDC 2165639 and 2165640) were collected at *T* = 100 K in a dry stream of nitrogen on a Bruker Kappa APEX II diffractometer system using graphite-monochromatized Mo-*K*α radiation (*λ* = 0.71073 Å) and fine sliced *φ*- and *ω-*scans. Data were reduced to intensity values with SAINT and an absorption correction was applied with the multi-scan approach implemented in SADABS [23]. The structures were solved by the dual space method implemented in SHELXT [24] and refined against *F*^2^ with SHELXL [25]. Non-hydrogen atoms were refined with anisotropic displacement parameters. The H atoms were placed in calculated positions and thereafter refined as riding on the parent C atoms. **2** was refined as an index 2 twin by pseudo-merohedry (monoclinic structure with pseudo-*oP* lattice). Molecular graphics were generated with the program MERCURY [26].

## Supplementary Information

Below is the link to the electronic supplementary material.

Supplementary file1 (PDF 347 KB)
