# Supplementary material for: Synthesis and characterization of cobalt SCS pincer complexes
Source: Monatsh Chem. 2022 Jul 16;153(7-8):545–9. doi: 10.1007/s00706-022-02949-1 (PMC9360111; doi:10.1007/s00706-022-02949-1)
Supplement: Supplementary file 1 — Supplementary file1 (PDF 347 KB) [file 706_2022_2949_MOESM1_ESM.pdf]

Supporting Information

Synthesis and Characterization of Cobalt SCS Pincer Complexes

Jan Pecak[1] ● Matthias Käfer[1] ● Sarah Fleissner[1] ● Werner Artner[2] ● Karl Kirchner[1 *]

[1] Institute of Applied Synthetic Chemistry, Vienna University of Technology, Getreidemarkt 9/163-AC, 1060 Wien, Austria.

[2] X-Ray Center, Vienna University of Technology, Getreidemarkt 9/163-AC, 1060 Wien, Austria.

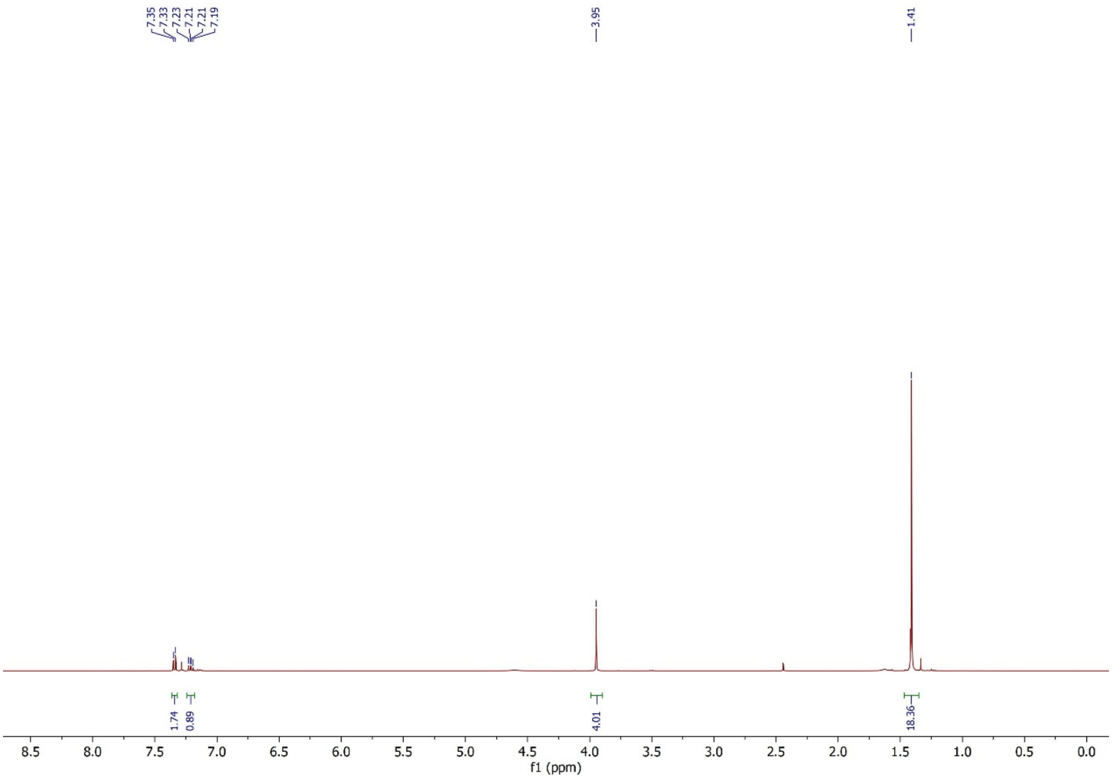

SI 1. ^{1}H NMR spectrum of the ligand S(C-Br)S^{CH2}-*t*Bu.

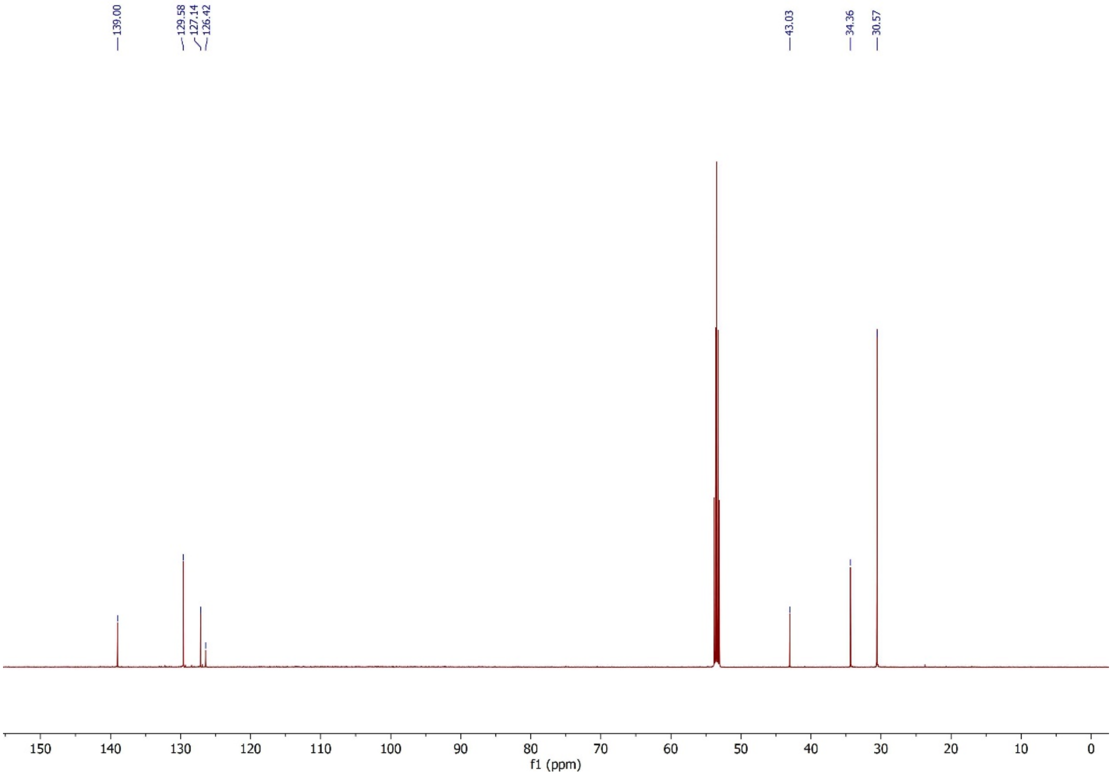

SI 2. ^{13}C{^{1}H} NMR spectrum of the ligand S(C-Br)S^{CH2}-*t*Bu.

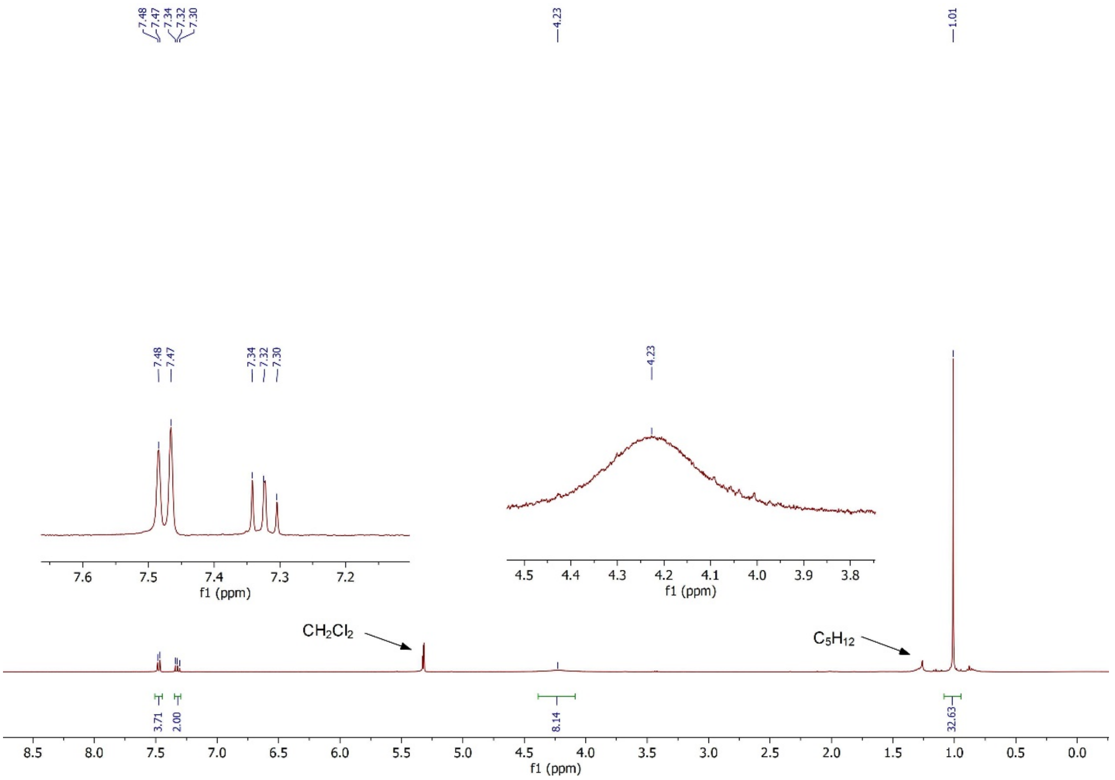

SI 3. ^{1}H NMR spectrum of cationic complex [Co(SCS^{CH2}-*t*Bu)$_2$] (**3**).

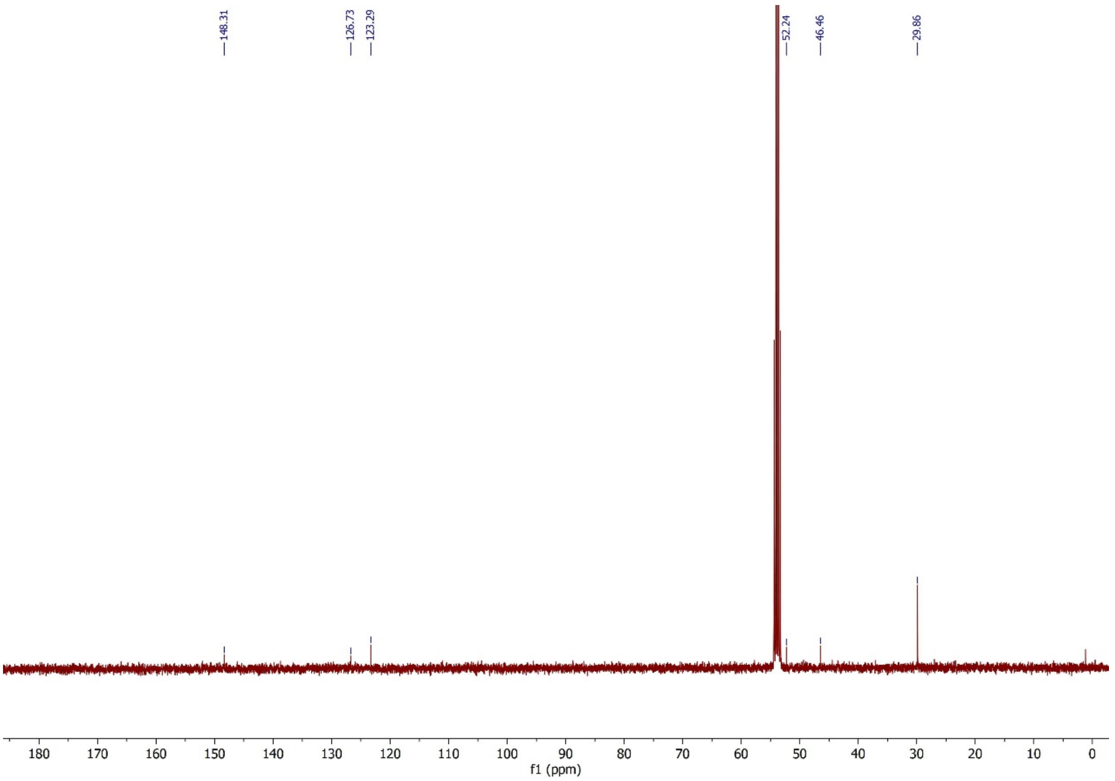

SI 4. $^{13}C\{^{1}H\}$ NMR spectrum of cationic complex [Co(SCS^{CH2}-*t*Bu)$_2$] (**3**).

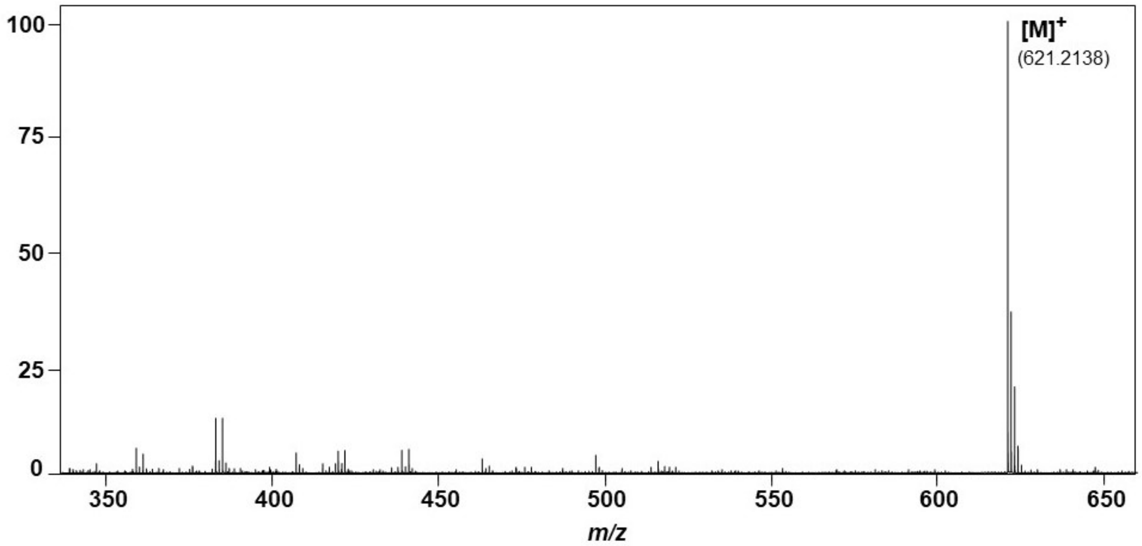

SI 5. HR-MS spectrum of cationic complex [Co(SCS^{CH2}-*t*Bu)$_2$] (**3**).

XYZ coordinates for optimized complex 1 (DFT)

C	1.722294000	1.491635000	22.672445000
S	3.818119000	-0.427361000	22.356680000
Co	3.076929000	1.507283000	21.275480000
C	0.509680000	2.222668000	22.539835000
S	1.920100000	3.424036000	20.553159000
C	-0.451502000	2.240614000	23.568987000
H	-1.383354000	2.817989000	23.447749000
S	4.561276000	2.819753000	22.508326000
C	-0.235478000	1.504656000	24.747655000
H	-0.992385000	1.507315000	25.547586000
S	1.986459000	0.212934000	19.657980000
C	1.925027000	0.773154000	23.883496000
C	0.950417000	0.765607000	24.901139000
H	1.120157000	0.190248000	25.826596000
C	3.239450000	0.046275000	24.049735000
H	3.168369000	-0.844366000	24.706824000
H	4.026312000	0.721591000	24.455703000
C	1.531376000	3.494022000	18.754390000
H	1.165424000	2.488522000	18.460888000
H	0.707028000	4.225928000	18.621547000
C	0.272334000	2.925019000	21.226443000
H	-0.397262000	3.806263000	21.305300000
H	-0.158565000	2.222583000	20.477103000
C	2.772201000	3.885845000	17.959705000
H	2.525428000	3.958136000	16.880804000
H	3.577040000	3.134672000	18.082545000
H	3.166643000	4.870600000	18.283815000
C	5.640534000	-0.454505000	22.569426000
H	6.018886000	-0.494359000	21.527205000
H	5.939302000	0.532379000	22.979121000
C	6.145678000	-1.619976000	23.412518000

H	5.818488000	-2.592457000	22.992028000
H	5.772766000	-1.561110000	24.455126000
H	7.255379000	-1.616910000	23.454774000
C	4.499260000	1.552833000	19.948445000
C	4.569839000	0.592094000	18.902779000
C	6.584008000	1.633463000	18.004166000
H	7.388049000	1.665541000	17.252249000
C	5.590371000	0.641180000	17.932403000
H	5.619346000	-0.103619000	17.118839000
C	6.547183000	2.585080000	19.038845000
H	7.324173000	3.366387000	19.092943000
C	5.383938000	3.615171000	21.055870000
H	4.697329000	4.429261000	20.728669000
H	6.354336000	4.066098000	21.346438000
C	5.509222000	2.553564000	19.989438000
C	3.519864000	-0.491191000	18.894849000
H	3.814984000	-1.343971000	19.548263000
H	3.298693000	-0.890701000	17.883882000
C	3.743191000	4.244595000	23.325903000
H	3.108838000	4.743184000	22.564089000
H	3.054663000	3.759420000	24.048348000
C	4.713483000	5.204361000	24.003838000
H	5.383635000	5.694439000	23.268200000
H	5.349095000	4.679584000	24.745608000
H	4.158195000	6.007643000	24.532170000
C	1.204324000	-1.329871000	20.290486000
H	1.072668000	-2.016008000	19.427574000
H	1.920225000	-1.787777000	21.004639000
C	-0.122547000	-1.005539000	20.968314000
H	-0.823322000	-0.503337000	20.269508000
H	0.032185000	-0.341756000	21.841663000
H	-0.606952000	-1.937722000	21.323596000
